# Ustekinumab in the Treatment of Crohn’s Disease—A Narrative Review on Clinical Efficacy and Safety Profile

**DOI:** 10.3390/pharmacy13030073

**Published:** 2025-05-21

**Authors:** Dawid Piecuch, Edyta Hańczyk, Szymon Kopciał, Natalia Pawelec, Weronika Mazur, Karolina Kornatowska

**Affiliations:** Faculty of Medicine, Kazimierz Pułaski University of Radom, 26-601 Radom, Poland; edyta.hanczyk25@gmail.com (E.H.); szymon.kopcial@gmail.com (S.K.); jancynatalia@gmail.com (N.P.); nikaaa665@gmail.com (W.M.); kkornatowska47@gmail.com (K.K.)

**Keywords:** Crohn’s disease (CD), ustekinumab (UST), immunomodulatory treatment, interleukins IL-12, IL-23, clinical remission (CR), adalimumab, infliximab, risankizumab (RZB), UST-biosimilar formulations

## Abstract

Crohn’s disease (CD) is a chronic inflammatory disorder of the gastrointestinal tract that leads to significant deterioration in patients’ quality of life. Biologic therapy, including the use of ustekinumab (UST), is a modern approach to treating the moderate to severe form of CD, especially in patients refractory to traditional treatments. UST, which acts as an interleukin IL-12 and IL-23 antagonist, has shown high efficacy in reducing inflammation, improving quality of life, and promoting mucosal regeneration and fistula healing. However, the use of biologic therapies, such as UST, has challenges related to the timing of treatment and patient response, including the problem of immunogenicity. To determine the clinical efficacy and safety profile of UST in the treatment of CD, a review of the literature published in the PubMed database over the last 5 years was conducted. After excluding articles that did not meet the inclusion criteria, we analyzed 42 clinical studies. The review discusses the available data on the efficacy and safety of UST, as well as its comparison with other biologic therapies, such as infliximab and adalimumab. UST, although not significantly greater to adalimumab, has lower immunogenicity and higher treatment retention. The therapeutic value of UST is also confirmed by biosimilars such as ABP 65 and FYB202, which show comparable efficacy and safety profile. The analysis of predictive biomarkers, such as serum drug levels and baseline eosinophil levels, could be an important element in the future personalization of CD treatment. The review’s findings point to the importance of further research to improve the tailoring of therapies to individual patients and improve long-term treatment outcomes.

## 1. Introduction

Crohn’s disease (CD) is a chronic, progressive inflammatory disorder of the gastrointestinal tract, primarily manifested by abdominal pain, chronic diarrhea, and weight loss, which significantly reduces patients’ quality of life [1]. As a component of inflammatory bowel disease (IBD), CD, along with ulcerative colitis (UC), is characterized by periods of exacerbation and remission, and can lead to serious complications such as intestinal strictures, fistulas, and risk of neoplasia [2]. CD is also often accompanied by extraintestinal manifestations (EIMs), which occur in almost half of patients with the disease. EIMs can affect a variety of systems, such as the joints, skin, eyes, liver, and hematologic system (e.g., anemia), and less commonly, the kidneys and lungs [3]. These symptoms can significantly reduce patients’ quality of life and require comprehensive treatment, which includes therapy targeting the gastrointestinal tract as well as symptomatic treatment and targeting extraintestinal manifestations [4,5,6,7,8]. Initially, it was adalimumab, a fully human high-affinity monoclonal antibody, that was the third TNF-α inhibitor to be introduced and, along with infliximab, significantly changed the treatment of moderate to severe CD [9]. In contrast, in recent years, the development of biologic therapies significantly improved treatment options for patients, especially those refractory to traditional treatments. The use of biologic therapies allows for the more effective control of chronic inflammation and a reduction in the number of hospitalizations associated with disease exacerbations. One of the modern drugs used to treat the disease is UST, a human monoclonal antibody of the IgG1 kappa (IgG1κ) class that acts as an antagonist of the p40 subunit of the interleukins IL-12 and IL-23, approved for the treatment of IBD [10,11]. As an interleukin inhibitor, UST has shown high efficacy in reducing inflammation, and its action on interleukins IL-12 and IL-23 contributes to the modulation of the immune response, which is crucial in the treatment of CD [11]. Current indications for UST include plaque psoriasis, psoriatic arthritis, CD, and UC [10,11,12,13,14,15]. Among interleukin IL-12 and IL-23 inhibitors, such as UST, guselkumab (GUS), tildrakizumab (TIL), and risankizumab (RZB), only RZB and UST were used to treat moderate to severe CD [5]. However, despite the widespread use of biologic therapies in the treatment of IBD, not all patients respond to treatment. When using USTs in pediatric patients with CD, there is an even greater need for the precise selection of appropriate therapy [16,17]. When using UST, the key factor is the decision to implement it at the right time and the optimal moment to start therapy should be determined by analyzing the severity of symptoms and the patient’s response to previous treatment. This review paper presents the available data on the efficacy and safety of USTs in the treatment of CD, with a focus on the results of clinical trials and long-term observations. Potential predictive biomarkers will also be analyzed that could support therapeutic decisions in the future, enabling the better tailoring of therapies to individual patients [4,5,6,7,8,9,16,17,18,19,20,21,22,23,24,25,26,27,28,29,30,31,32,33,34,35,36,37,38,39]. The results of these studies may contribute to a more personalized approach in the treatment of CD, thereby improving therapy outcomes and patients’ quality of life.

## 2. Materials and Methods

**Figure 1:**
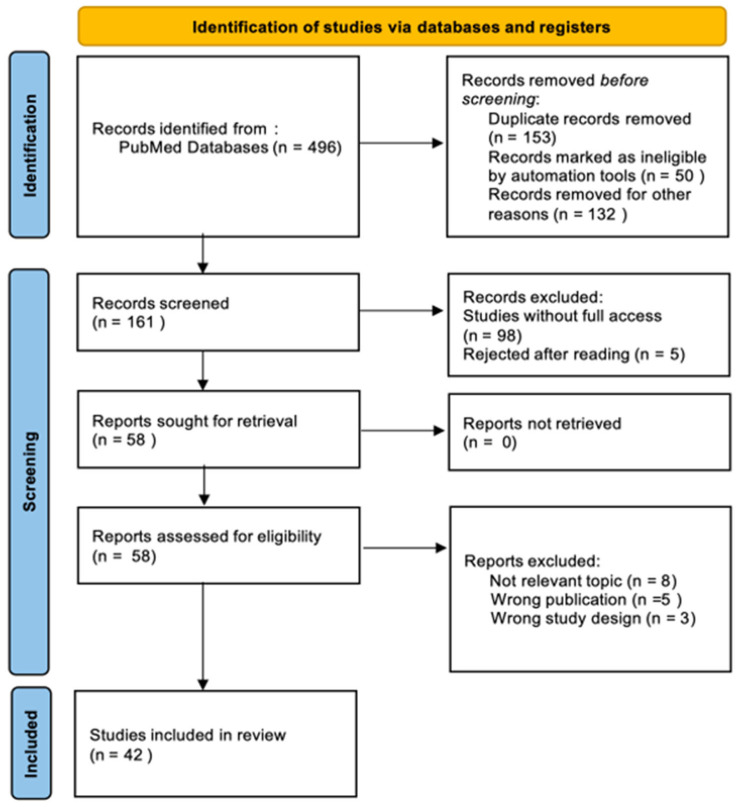
Preferred Reporting Items of Systematic Review and Meta-analyses (PRISMA) flow diagram.

To evaluate the clinical efficacy and safety profile of ustekinumab in the treatment of CD, we conducted a comprehensive literature search of the PubMed database. The search was performed on 25 March 2025 to ensure the inclusion of the most recent and relevant studies. This review was performed in accordance to the PRISMA (Preferred Reporting Items for Systematic Reviews and Meta-Analyses) guidelines. The following search terms were used to identify relevant studies: (“Ustekinumab”) OR (“Crohn’s Disease”). Only full-text articles with free access, categorized under clinical trials, randomized controlled trials, and books and documents, were considered for inclusion. Due to the variation in study designs, only those that evaluated the efficacy and safety of UST in comparison to other therapies or placebo, or those that provided real-world evidence on its long-term use, were included in the final analysis. Four authors independently participated in the study selection and data extraction process. Each search result was screened by at least two reviewers based on titles and abstracts. Full texts of potentially eligible studies were then reviewed to determine final inclusion. Data extraction was conducted using a standardized form, and discrepancies were resolved through group discussion to ensure consistency and accuracy. During the literature search process, special attention was taken to identify potential duplicates that could arise from different databases or multiple publications of the same results in different journals. The search was designed to identify such cases by comparing titles, authors, publication dates, and DOI (digital object identifier) numbers. Figure 1 presents a flowchart of the study selection process, highlighting the articles that met the inclusion criteria.

## 3. Results and Discussion

Table 1 provides a concise summary of the main studies included for qualitative analysis, outlining their key characteristics in the context of UST treatment for CD. This overview forms the basis for further discussion on clinical efficacy and safety, highlighting the diversity of study designs and patient populations.

Table 2 summarizes the main findings from studies evaluating UST in the treatment of CD. Organized by thematic subsections, it presents synthesized conclusions and key outcomes with the corresponding citations. The format facilitates easy tracking and comparison of evidence across domains such as clinical efficacy, safety, dosing strategies, fistula response, extraintestinal manifestations, and quality of life.

### 3.1. Efficacy of Ustekinumab in the Treatment of CD

The multicenter study evaluated the actual efficacy of UST after 52 weeks of treatment. Among the patients, 52% achieved the primary objective with a combination of clinical and endoscopic response, with age proving to be a significant independent predictor. 15 patients achieved an endoscopic response, 9 patients achieved endoscopic remission, and 12 patients had improvement in intestinal mucosal healing assessed by endoscopy. Healing of intestinal fistulas was found in all patients, including 33.35% of those on multiple biologics. Extra-intestinal manifestations (EIMs) dropped significantly, 20.2% after 52 weeks [7]. The study by Jia-Yin Yao et al. proved that minimal UST reflects the success of UST therapy. After UST treatment, 19/20 subjects experienced a significant decrease in mean CDAI values from 220.5 ± 58.8 to 92.4 ± 48.5, and simple endoscopic score for CD (SES-CD) decreased from 11.2 ± 6.1 to 4.4 ± 4.2 at week 16 of treatment. The researchers showed an average minimum UST concentration threshold of 1.12 μg/mL. Achieving the aforementioned drug concentration was associated with a higher endoscopic remission rate compared to those with lower values (a difference of 58.9%). Concentrations exceeding 1.12 μg/mL compared to patients with lower concentrations were associated with better clinical response, clinical remission, endoscopic response, and the return of CRP to values within the normal range (difference 1.1%, 12.2%, 34.4%, 26.7%) [18]. Researchers Neeraj Narula et al. showed that there was no significant difference in the percentage of patients taking UST who developed EIMs compared to those taking placebo. After 6 weeks of UST treatment, only 2.2% more EIMs were observed compared to placebo. When EIM was assessed after one year of treatment, EIM was found to have decreased in both treatment groups, but only the UST-treated group had 100% resolution of symptoms. The drug reduced the onset of EIM in 527 patients by 35% at week 6, and by 72% in 146 patients at week 52. The greatest improvement over the initial number of patients occurred in arthritis/joint pain (week 6–32%, week 52–69%). In contrast, erythema nodosum and iritis/vasculitis achieved very high rates of symptom resolution at week 52 (100% and 87.5%) [6].

Almradi A. et al., in their study, showed that UST is effective in inducing and maintaining clinical remission in patients with moderate to severe CD. The UNITI-1 and UNITI-2 studies evaluated the efficacy of UST in patients who had previously failed to respond to TNF antagonist therapy (UNITI-1) and in those who had failed conventional treatments such as immunosuppressants or glucocorticosteroids (UNITI-2). In the UNITI-1 study, a clinical response at week 6 was achieved by 34.3% of patients in the 130 mg UST group and 33.7% in the ~6 mg/kg group, compared to 21.5% in the placebo group (*p* = 0.002; *p* = 0.003). In the UNITI-2 trial, 51.5–55.7% of patients achieved a clinical response, which was significantly higher than in the placebo group (28.7%; *p* < 0.001). At week 44, clinical remission was achieved by 53.1% of patients receiving UST every 8 weeks and 48.8% every 12 weeks, and 35.9% in the placebo group (*p* = 0.005 and *p* = 0.04 compared to placebo). Endoscopic and histologic responses were more frequent in the UST group, especially with dosing every 8 weeks. CD had a slightly higher rate of side effects than psoriasis and psoriatic arthritis. The risk of infection was not increased, and anti-drug antibodies [ADAs] were detected in 2.3–4.6% of patients [19].

The IM-UNITI and long-term extension (LTE) study by William J. Sandborn et al. evaluated the 5-year safety and efficacy of subcutaneously administered UST in patients on CD therapy. Of the patients who responded to treatment after 8 weeks, they were randomized to groups receiving placebo, UST 90 mg every 12 weeks (q12w) or UST 90 mg every 8 weeks (q8w). After completing the 44th week, 298 patients continued to participate in LTE. At week 44, clinical remission was achieved by 77.4% of patients in the q12w group, 84.1% in q8w, and 63.4% in the group in which the dose was adjusted earlier. Patients receiving placebo discontinued treatment at week 44 after the study was unblinded. Among patients treated with UST and continuing to participate in LTE, 52.3% (124/237) completed treatment after 5 years. The most common reasons for discontinuing therapy were adverse effects, lack of efficacy and withdrawal of consent. Discontinuation rates were similar between the q12w (46.4%) and q8w (41.5%) groups. After 5 years, clinical remission was achieved by 28.7% of patients receiving UST every 12 weeks (q12w) and 34.4% receiving it every 8 weeks (q8w), with a rate of 44% for q8w among those not previously treated with TNF antagonists. Both UST doses performed similarly, with q8w showing slightly better results, especially in patients without prior TNF antagonist therapy [20]. In a prospective study involving 221 patients with CD, almost all of whom had been previously treated with anti-TNF drugs (98.6%) and almost half with vedolizumab (46.6%), ustekinumab showed moderate clinical efficacy and a favorable safety profile in real-world clinical settings. The percentages of clinical remission without corticosteroids were 38.2% at week 24 and 37.1% at week 52 of follow-up [38]. Researchers reached similar conclusions when they conducted a long-term safety analysis of UST. In clinical trials involving 2575 patients and 4826 patient-years of follow-up, the incidence of serious adverse events, such as heart disease or malignancies, was similar for placebo and UST. Opportunistic infections, tuberculosis and malignancies were rare, and the lack of reported cases of lymphoma or posterior reversible encephalopathy syndrome further confirms the safety of the therapy. These results indicate that UST remains a well-tolerated therapeutic option [21]. Based on the STARDUST trial, Peyrin-Biroulet L. et al. evaluated the efficacy of UST in LTE. At week 104, clinical remission was achieved by 50.2% of all randomized patients and 68.4% of patients continuing LTE, and endoscopic response was achieved by 28.9% and 39.3%, respectively. The therapy was well tolerated, with no new safety signals. Flexible dosing allowed treatment efficacy to be maintained, with 23.5% of patients requiring escalation and 19.7% requiring the de-escalation of therapy [22]. Colombel J.F. et al., in their study, analyzed the early efficacy of therapy during the first 2 weeks after the induction infusion and the cumulative results up to week 16 after the first subcutaneous injection of UST at a dose of 90 mg. Patients showed a significant improvement in overall well-being as early as day 1 after infusion (*p* < 0.05) compared to placebo. Clinical response and remission rates were significantly higher than in the placebo group. By week 16, cumulative clinical remission rates were 55.5% in patients with no prior failure of biologic therapy and 24.1% in patients with such a history. By week 44, up to 66.7% of patients remained in clinical response. In patients without a history of biological failure, the response appeared faster, while in patients with a history of biological failure, about 20% achieved a response only after SC injection at week 8 [23]. This large, prospective, multicenter real-world study conducted across 20 Swedish hospitals assessed the long-term effectiveness and safety of ustekinumab in 114 adult patients with active CD, most of whom had previously failed multiple biologics. Clinical remission (HBI ≤ 4) was achieved in 32% and 29% of patients at weeks 52 and 104, respectively, with corresponding clinical response rates of 36% and 29%. Treatment retention was high (70% and 61%), and ustekinumab led to significant improvements in inflammatory markers (CRP, fecal calprotectin) and HRQoL. Serious adverse events were infrequent (4.4%), and no malignancies were reported. Despite this, ustekinumab showed durable clinical benefits and improved HRQoL, supporting its role in routine clinical care for moderate-to-severe CD. The robust design and systematic data collection make this one of the most comprehensive long-term prospective evaluations of ustekinumab to date [39]. After 52 weeks, 64% of patients with active disease (HBI > 4) achieved clinical remission, and calprotectin and CRP levels normalized in 54% and 37% of patients, respectively. Treatment was terminated in 27.5% of patients, and adverse effects occurred in 60 patients. Patients with less exposure to prior anti-TNFα therapies and with disease localization in the ileum had better clinical outcomes. Advanced endoscopic lesions were associated with a poorer response to therapy [42]. In the context of treatment safety, it is important to consider the differences in therapeutic response according to patient subgroups, such as age, gender, and the presence of comorbidities. Studies indicate that certain groups, such as elderly patients or those with heart disease, may be at higher risk of certain side effects. There is a risk that the immune response to the drug could lead to the development of neutralizing antibodies, which could affect the effectiveness of treatment and patient safety.

### 3.2. Predictive Factors of Response to Therapy and Minimum UST Levels

The post hoc analysis of the UNITI-1/2 study by Emily C.L. Wong et al. focused on determining the effect of UST iv 130 mg/6 mg per kilogram of body weight in the determining predictive factors of CD disease. It is noteworthy that the subjects were allowed to take other adjunctive medications as long as their dose was kept at the same level. Among the prognostic factors tested, shorter disease duration (<5 years for remission and <1 year for clinical response) proved to be a key predictor, increasing the likelihood of a positive response to therapy [24]. Emily C.L. Wong et al. also showed that, in a group of CD patients receiving UST, those responding positively to therapy had a significantly higher initial eosinophil count compared to those in whom treatment had no effect (0.18 × 10^9^ vs. 0.12 × 10^9^). Trends in serum eosinophil values at 0, 2, 8 weeks of the study were evaluated, yielding the following values in UST responders: 0.18, 0.20, 0.14. When eosinophil levels in the study increased by more than 10% from the start of the study, the chance of response by week 8 was lower than in patients with less than a 10% increase or no increase (*p* = 0.048). When eosinophil levels decreased by more than 10% since the start of the study, the chance of a clinical response at week 8 was significantly higher compared to patients with no reduction in eosinophils [25]. Vince B. C. Biemans et al. showed that the use of a dosing regimen every 8 weeks (q8w) was associated with a significantly lower discontinuation rate compared to a q12w regimen (20.0% vs. 42.6%, *p* = 0.01), although this did not translate into a significantly higher rate of corticosteroid-free clinical remission at week 52 (46.3% vs. 34.6%, *p* = 0.20). There was also no observed benefit from the combination therapy of UST with immunosuppressants compared to monotherapy (40.6% vs. 36.0%, *p* = 0.64) [38]. UST shows good efficacy and safety profile in both short- and long-term use in patients with refractory CD. The study included 463 patients with CD. The vast majority, 96.5% (447 patients), had been previously treated with biologic drugs, and more than 30% (141 patients) had been treated with three or more such agents. In addition, 35.2% of patients were using concomitant immunosuppression, and nearly half (47.1%) had undergone at least one abdominal surgery. At week 16 of UST treatment, clinical remission was achieved in 56% of patients and response to therapy in 70%. Dose intensification was necessary in 26.1% of patients, almost a quarter of whom (24.8%) could not later return to a lower dose. After an average of 15 months, 77% of patients (356 patients) continued treatment [41]. The study by Jia-Yin Yao et al. proved that minimal UST reflects the success of UST therapy. After UST treatment, 19/20 subjects experienced a significant decrease in mean CDAI values from 220.5 ± 58.8 to 92.4 ± 48.5, and a simple endoscopic score for CD (SES-CD) decreased from 11.2 ± 6.1 to 4.4 ± 4.2 at week 16 of treatment. The researchers showed an average minimum UST concentration threshold of 1.12 μg/mL. Achieving the aforementioned drug concentration was associated with a higher endoscopic remission rate compared to those with lower values (a difference of 58.9%). Concentrations exceeding 1.12 μg/mL, compared to patients with lower concentrations, were associated with better clinical response, clinical remission, endoscopic response, return of CRP to values within the normal range (difference 1.1%, 12.2%, 34.4%, 26.7%) [18]. In a study by Emily C.L. Wong et al. that looked at the efficacy of UST on the clinical remission of CD depending on body mass index (BMI), it was shown that, after 308 days of treatment, drug levels were significantly lower in obese patients (2.98 µg/mL) compared to overweight (4.84 µg/mL), normal weight (4.43 µg/mL), and underweight (4.43 µg/mL) patients. However, there was no correlation between the significance and frequency of improvement in patients’ conditions depending on their BMI. In underweight, normal BMI, overweight, obese patients, CR was as follows: 68%, 51%, 45%, 55% [26]. Mark T. Osterman et al. investigated whether the ileal microvessel length (MVL) reflects the clinical status of patients taking UST (N = 70). They found that patients treated with UST showed greater clinical response (40:13) and clinical remission (26:8) compared to the group taking placebo. Patients with normal ileal microvessel length (>1.7 µm) had the highest efficacy of UST therapy (85% vs. 20%) [27].

### 3.3. Clinical Implications of UST Therapy

A. Tursi et al. studied the clinical effects of UST therapy in a group of 194 patients, 76% of whom had previously taken anti-TNFα drugs, while 24% were treated with vedolizumab and anti-TNFα, and as many as 91% of the subjects were taking steroids at the same time. The withdrawal of the clinical symptoms of CD disease after 4 months was observed in 75% of the subjects, while after 6 months, this value decreased to 70%. Endoscopic symptoms withdrew in more than 50% of the subjects. During the study, it was noted that CRP and fecal calprotectin (FC) decreased during UST therapy, and FC exceeding 200 µg/g correlated with lack of disease remission. TEAEs occurred in only 3% of those treated [28].

A clinical study by William J. Sandborn et al. involving 2574 patients, including six independent studies, showed that the number of TEAEs per 100 patient–years was 118.32 compared to placebo’s 165.99. Among the most common effects of therapy were serious adverse events (27.50 placebo:21.23 UST), infections (80.31:64.32), serious infections (5.53:5.02), headache, fatigue, nasopharyngitis, upper respiratory tract infection, or fever. The safety profile of UST in the combined inflammatory bowel disease patient population at one year was favorable and not significantly different from placebo [29]. Treatment-emergent adverse events (TEAEs) occurred in 69.2% of participants, among which 15.4% of cases were considered severe, but no serious TEAEs or deaths were observed [7].

In a study by Bruce E. Sands et al., clinically significant infections were reported in 4/191 taking USTK and 5/195 taking adalimumab. Among the most common adverse effects of UST therapy were infections n-65, abdominal pain n-24, headache n-22, nasopharyngitis n-14, joint pain n-12, upper respiratory tract infection n-12, and diarrhea n-10. In contrast, the most common adverse effects of adalimumab therapy were infections n-79, nasopharyngitis n-19, abdominal pain n-16, joint pain n-16, upper respiratory tract infection n-15, and injection site erythema n-13 [8]. Headache (n = 210) was the most common adverse event of UST biosimilar substitute therapy [30].

The STARDUST study, on the other hand, focused on evaluating the effect of UST on intestinal inflammation in patients with moderate to severe CD. The study focused on whether a “treat-to-target” (T2T) treatment strategy, which includes early endoscopy, the regular monitoring of biomarkers and clinical symptoms, and the intensification of therapy if inflammation persists, is more effective in improving endoscopic outcomes than standard care (SoC) based on clinical symptoms. The study included 498 patients, 440 of whom were randomly assigned to either T2T (n = 219) or SoC (n = 221) [22,31,32,33]. The researchers evaluated the treatment outcomes after 48 weeks, finding no significant differences between groups in endoscopic response (38% vs. 30%), endoscopic remission (11% vs. 15%), mucosal healing (14% vs. 17%), and clinical remission (62% vs. 70%). In contrast, clinical response was significantly lower in the target treatment group (68% vs. 78%). Overall endoscopic, clinical, and biomarker outcomes did not differ significantly between groups. The most commonly reported side effects were nasopharyngitis, abdominal pain, joint pain, and headache, occurring with similar frequency in both groups [32].

On the basis of the above study, Kucharzik T. et al. wanted to assess early ultrasound response and trans-wall remission using a non-invasive imaging modality, intestinal ultrasonography (IUS), for evaluation. At week 48, the IUS response rate was 46.3%, and trans-wall remission was achieved in 24.1% of patients. The results of this study confirmed a moderate concordance between IUS response and endoscopic findings and biomarkers, highlighting the importance of IUS as an adjunct to traditional methods of CD evaluation. Higher transmural remission rates were observed in the colon than in the terminal ileum, which may be due to the pathophysiological differences between these regions. Patients previously untreated with biologics showed a stronger response to therapy [31]. Julian Panes et al. assessed the quality of life of patients treated with UST up to the 104th week on the basis of the multicenter STARDUST study. They assessed work productivity, activity impairment (WPAI) and health-related quality of life (HRQoL) using several questionnaires. No significant differences were found between the T2T and SoC strategies, as both were effective. UST improved HRQoL, reduced fatigue, and increased patient productivity for 2 years [33].

In contrast, focusing on the same study, Peyrin-Biroulet L. et al. evaluated the efficacy of UST in prolonged long-term treatment. At week 104, clinical remission was achieved by 50.2% of all randomized patients and 68.4% of patients continuing LTE, and endoscopic response by 28.9% and 39.3%, respectively. The therapy was well tolerated, with no new safety signals. Flexible dosing allowed treatment efficacy to be maintained, with 23.5% of patients requiring escalation and 19.7% requiring the de-escalation of therapy [22].

### 3.4. Quality of Life in Patients Undergoing UST Therapy

Treatment with ustekinumab significantly improves the quality of life in patients with Crohn’s disease (CD), as confirmed by the results of the multicenter STARDUST trial. Julian Panés et al. evaluated parameters such as work productivity, limitations in daily activities (WPAI), and HRQoL using standardized questionnaires. After two years of therapy, a significant improvement in these outcomes was observed, regardless of the treatment strategy applied (treat-to-target vs. standard care) [33]. Moreover, according to the data presented by Turner D. et al. in the UniStar study, long-term UST therapy in children with CD (up to 240 weeks) also led to a marked improvement in quality of life, which coincided with the achievement of clinical remission and normalization of inflammatory markers [16]. The reduction in disease symptoms, decreased fatigue, and improved functioning in both professional and social life are key components contributing to the subjective perception of improved quality of life in patients treated with UST. The median subjective level of well-being steadily increased in those who continued therapy, reaching significantly higher values at weeks 52 and 104 compared to baseline. Importantly, median well-being was noticeably lower in patients who discontinued therapy, further confirming the positive effect of continuing UST therapy on quality of life [40]. In the study by Anders Forss et al., a statistically significant improvement in HRQoL was noted among the clinical parameters assessed. This improvement was correlated with a reduction in clinical symptoms (decrease in HBI and CRP levels) and achievement of clinical response (36% at week 52 and 29% at week 104) and remission (32% and 29%, respectively) [39].

Table 3 presents a summary of adverse reactions reported in the studies included in this narrative review, providing information on the safety profile of UST for the treatment of CD. The summary provides a comparison of the incidence and nature of adverse effects, allowing a more complete assessment of the balance of benefits and the risks of the drug under different clinical conditions.

### 3.5. Comparison with Other Biologic Therapies

#### 3.5.1. UST and Adalimumab

Researchers Prof. Bruce E. Sands et al. compared the efficacy of UST (0–6 mg/kg iv then 90 mg sc. every 8 weeks) and adalimumab (0–160 mg then 90 mg sc. every 8 weeks) in patients showing moderate to severe CD disease activity (CDAI = 220–450). Important inclusion criteria were previous unresponsiveness to primary therapies and the presence of an ulcer visualized by gastrointestinal endoscopy. After one year of clinical remission, CDAI < 150 was assessed to be comparable in both groups. There were 4% more patients treated with UST 124/191 that showed a remission of CD symptoms. UST treatment appears to be better tolerated by patients as 17 more patients discontinued therapy before week 52 compared to those taking adalimumab. The withdrawal of endoscopic lesions was observed in 29 patients receiving UST and 32 treated with adalimumab. In patients receiving UST, only 2% developed an immune response to the drug compared to 74% receiving adalimumab [8]. A post hoc analysis by Neeraj Narula et al. compared therapeutic outcomes in patients responding early to the treatment with UST and adalimumab (up to 8 weeks of treatment) with late responders (8–16 weeks of treatment) and non-responders (>16 weeks). After one year of treatment time with UST (n = 187) and adalimumab (n = 186), patients responding up to week 8 and 16 had comparable CD activity indices (CDAIs). Late responders showed a decrease in inflammatory C-reactive protein (CRP) exponents by week 8 which was not observed in non-responders. The clinical response after one year of treatment with both drugs was similar in early and late responders [36]. In the group of CD patients receiving UST, those responding positively to therapy had significantly higher initial eosinophil counts compared to those in whom treatment had no effect, while no significant differences in eosinophil counts were observed in CD patients treated with adalimumab [25].

#### 3.5.2. UST and Risankizumab

Peyrin-Biroulet L. et al. conducted a study involving 527 patients with moderate-to-severe CD. Patients were eligible for the study because they had not responded sufficiently to anti-TNF therapy or had experienced unacceptable side effects. They were randomly assigned to treatment with RAB or UST at standard doses for 48 weeks. Two endpoints included clinical remission at week 24 and endoscopic remission at week 48 to assess the efficacy of long-term treatment of patients with these drugs. At week 24, UST was no worse than RZB in terms of clinical remission (58.6% vs. 39.5%), but at week 48, RZB was greater in terms of endoscopic remission (31.8% vs. 16.2%; *p* < 0.001). At week 48, hospitalization rates were lower in the RZB group. Both drugs had an acceptable safety profile, with no new risks [4]. Similar conclusions were reached by Dubinsky M. et al., who conducted a study on comparing the efficacy of the two drugs. Patients received the following drug doses during the induction phase of treatment: one dose of UST 6 mg/kg IV at week 0 or RZB 600 mg intravenously (iv) at weeks 0, 4, and 8. In contrast, during the maintenance phase of treatment, they received RZB 180 or 360 mg subcutaneously (SC) or UST 90 mg SC every 8 or 12 weeks for up to 52 weeks. The MAIC (matching-adjusted indirect comparison) method was used to adjust for differences between patient populations from the clinical trials of the two drugs. The results suggest that RZB shows better efficacy in inducing clinical and endoscopic remission compared to UST, especially in a patient population where prior biologic therapy has failed. During maintenance therapy, the superiority of RZB in terms of endoscopic remission has also been reported [5].

#### 3.5.3. UST and Infliximab

Narula N. et al. conducted a post hoc analysis of two large clinical trials comparing the efficacy and speed of infliximab and UST in patients with moderate-to-severe CD who had not previously received biologic therapy. Among 420 patients, at week 6, clinical remission was achieved by 44.9% of patients receiving infliximab and 37.9% of those receiving UST (aOR 1.22; 95% CI 0.79–1.89). Clinical response was achieved by 58.4% and 54.9% of patients, respectively (aOR 1.25; 95% CI 0.82–1.90). There were no significant differences in the normalization of FC levels. Results were consistent across all analyses [9]. The study by Wong E.C.L. et al. included 220 patients with CD not previously treated with biologics who responded to induction therapy in two clinical trial programs. They compared the efficacy of infliximab and UST in achieving one-year clinical remission (CR), CR without corticosteroids, FC normalization, endoscopic response, and endoscopic remission (ER). Results showed that one-year CR (60.0% for infliximab vs. 57.3% for UST; aOR 1.15; *p* = 0.681) and CR without corticosteroids (39.3% vs. 52.9%; aOR 0.58; *p* = 0.251) were similar between groups. However, infliximab was associated with a higher likelihood of endoscopic response (46.7% vs. 20.0%; aOR 3.59; *p* = 0.011) and endoscopic remission (33.7% vs. 13.3%; aOR 3.35; *p* = 0.038). FC normalization was similar in both groups. Results were consistent in propensity-matched population analyses [34].

#### 3.5.4. UST and Biosimilars Substitutes

Biosimilars like ABP 65 and FYB202 are regulated by the same agencies (FDA and EMA) and meet high standards of quality and safety. Their similarity to the reference products (including UST) means that they can be used as substitutes for the original therapies, offering the potential for reduced treatment costs. Further research into their safety, immunogenicity, and efficacy will help us better understand their role in treating inflammatory diseases, as well as optimizing costs within healthcare systems.

ABP 65 is a biosimilar to registered UST, which works by inhibiting interleukin-12 and interleukin-23. Vincent Chow et al. conducted a randomized controlled trial in which they administered sc 90 mg of UST or ABP 65 to healthy patients at one time. The administration of ABP65 was associated with the production of fewer ADA antibodies compared to UST, but in no case did this result in the appearance of adverse events (AEs) of therapy (ABP 65—12/78/15.4%, UST US—30/79/38.0%, UST EU—29/80/36.3%). The safety profile of both substances appears to be very similar and is associated with quantitatively similar adverse effects, not exceeding grade 2 (ABP 65—22/78, UST US—18/79, UST EU—29/80). The adverse event that was reported by patients most frequently was a headache, occurring in a total of 21 subjects. The researchers showed that the safety profile, immunogenicity and pharmacological similarity (PK) of USTs from the United States (US) and the European Union (EU) are similar to each other [30]. Another biosimilar UST whose safety and efficacy was studied by Sigrid Balser et al. is FYB202. The researchers compared FYB202’s UST to USTs approved in the US and the EU after 112 days, administering a total of 491 healthy subjects in a 1:1:1 ratio, 1 × 45 mg sc. The elimination half-life of the drugs was 20 and 19.18 days, respectively. Positive ADA after 112 days was observed in 32/164, 83/164, 69/163 subjects, respectively. In all groups, mild AEs occurred in 326 subjects, while moderate AEs occurred in 187 subjects. Similarly to the study by Vincent Chow et al., headache was the most common AE and occurred in 16.5%, 24.4%, and 17.8% of cases, respectively. Nasopharyngitis, which occurred in 13.4%, 12.2%, and 13.5% of cases, and erythema at the injection site, which occurred in 3.7%, 3.7%, and 6.1% of cases, were also frequent AEs [30,35].

### 3.6. Ustekinumab in Pediatric Patients

The UniStar study is a randomized trial evaluating the pharmacokinetics, efficacy, and safety of UST in pediatric patients with moderate to severe active CD. It included intravenous drug induction at various doses, followed by maintenance therapy with subcutaneous UST, and long-term follow-up of up to 240 weeks of treatment. The study included 44 patients aged 2–<18 years (≥10 kg) who were randomly assigned to one of two weight-dependent intravenous UST induction doses: 130 mg vs. 390 mg for patients ≥ 40 kg and 3 mg/kg vs. 9 mg/kg for patients < 40 kg. At week 8, all patients received a single subcutaneous maintenance dose: 90 mg (≥40 kg) or 2 mg/kg (<40 kg) [16,17]. In a study by Rosh J.R. et al., the results showed that serum UST levels were comparable to those obtained in the adult CD patient population, but children weighing less than 40 kg had lower levels of the drug, suggesting the need to adjust the dosing regimen for this group. The induction treatment with UST improved the efficacy outcomes at week 8, including clinical response and remission, and reduced the levels of inflammatory biomarkers [17]. In contrast, Turner D. et al. in the UniStar study focused on the efficacy and safety of UST in a long-term extension study. Patients (n = 34) who responded to UST treatment at week 16 were enrolled in the study. Patients received their last dose at week 240. Long-term maintenance therapy with UST in children with CD resulted in improved clinical remission and quality of life after one year of treatment. At week 48, remission was achieved in 41.2% of patients, and therapy reduced the levels of inflammatory biomarkers such as CRP, calprotectin, and fecal lactoferrin. Normalization of calprotectin was achieved by 36.8% of children. Most complications were disease-related, and the most common serious adverse event was CD exacerbation (n = 6). No significant antibodies to UST were detected, except in one case [16].

The following Table 4 compares the treatment of adults and children with CD using UST, focusing on various aspects such as the dosing regimen, efficacy, biomarkers, and safety outcomes. The information presented is based on the data from clinical trials conducted in both adult and pediatric populations, such as UNITI and UniStar studies.

From the comparison, it is evident that, while the basic treatment approach for ustekinumab is similar in both adult and pediatric populations, there are key differences. For example, in the pediatric population, the dosing regimen is weight-dependent, especially for children under 40 kg, with some adjustments required due to lower serum UST levels. Additionally, while both adult and pediatric populations show improvements in clinical outcomes, pediatric patients tend to have a higher rate of clinical remission and a more favorable safety profile, with fewer serious adverse events observed compared to adults.

## 4. Conclusions

Ustekinumab has demonstrated robust efficacy and safety in the treatment of CD, particularly in patients who previously failed biologic therapies such as vedolizumab or TNF-α inhibitors [28,41]. Real-world data confirm sustained clinical remission and response, a relatively low discontinuation rate (primarily due to loss of response), and meaningful improvements in HRQoL over both short- and long-term follow-up [22,33,39,40,41,42]. After two years of treatment, approximately one-third of patients achieved corticosteroid-free clinical remission [40], with remission rates ranging from 32.3% to 41.4% within the first year and 34.0% at week 104. UST promotes mucosal healing, fistula closure, and the alleviation of EIMs such as arthritis and erythema nodosum [6,7,40], although it does not significantly reduce the risk of new EIMs in the short term.

Individual factors may influence treatment response. Shorter disease duration has been linked to better outcomes [24], while BMI does not appear to impact efficacy [26]. Around 30% of biologic-naïve patients fail to respond to induction therapy, and nearly half of initial responders lose response over time, making drug durability and immunogenicity critical in therapeutic planning [34]. Certain biomarkers may support treatment selection. Higher baseline eosinophil counts [25], intestinal microvilli length greater than 1.7 μm [27], and drug levels exceeding 1.12 μg/mL [18] have been associated with improved clinical and endoscopic outcomes, including CRP normalization. Endoscopic remission was achieved in 38% of patients at one year, with a mean CDAI reduction of −149 compared to −59 in the placebo group [37].

UST has a favorable safety profile, with an incidence of adverse events comparable to placebo [19,20,29]. Common side effects include headache (6.7%), fatigue, nasopharyngitis, upper respiratory infections, and fever. In pediatric populations, UST also appears safe; although lower induction doses were linked to more frequent flares, no severe infections or malignancies were reported during four years of follow-up [16,17]. UST delivers early symptom relief and increasing efficacy through week 44 [23], and subcutaneous maintenance dosing should begin at week 8 regardless of initial clinical response. However, escalation strategies based on early biomarker and endoscopic response (as in the STARDUST trial) did not significantly outperform the standard treatment [32]. IUS has demonstrated high concordance with endoscopy for identifying lesion location, making it a useful monitoring tool [31]. The maintenance interval may influence treatment retention: dosing q8w was associated with fewer discontinuations than q12w, though this did not result in significantly improved corticosteroid-free remission at week 52 [38]. In comparison with adalimumab, UST showed similar efficacy in achieving clinical remission over one year in biologic-naïve patients, while demonstrating better treatment persistence and lower immunogenicity [8]. Both early and late responders reached comparable remission rates at one year, emphasizing the importance of not abandoning therapy prematurely [36]. Newer agents, particularly RZB, demonstrated superior outcomes to UST across several parameters, including clinical response, steroid-free remission, and mucosal healing, according to STRIDE-II criteria [4,5]. Despite the methodological limitations of cross-trial comparisons, these findings support more personalized therapeutic choices.

Infliximab and UST are both effective, though infliximab may offer advantages in achieving endoscopic remission. Clinical remission at week 6 was observed in 44.9% of infliximab-treated versus 37.9% of UST-treated patients (aOR 1.22), and endoscopic response at one year favored infliximab (46.7% vs. 20.0%, aOR 3.59) [9,34,42]. Biosimilar versions of UST such as ABP 65 and FYB202 have shown comparable efficacy and safety to the originator drug. ABP 65 demonstrated lower immunogenicity [30], and FYB202 produced similar outcomes in both efficacy and safety measures [30,35]. Taken together, current evidence supports UST as a valuable treatment option for CD, particularly in patients with prior biologic failure, while also acknowledging that agents like RZB may offer superior outcomes in select cases. These findings emphasize the need for individualized, biomarker-driven treatment strategies in optimizing outcomes for patients with CD, highlighting critical gaps in our understanding of drug durability, immunogenicity, and predictive biomarkers. Further research is needed to explore these factors and refine therapeutic decision making in clinical practice.

## 5. Limitations of the Review

This review focuses on studies published within the last five years, which may exclude earlier but still relevant research on ustekinumab’s long-term efficacy and safety. As a narrative review, this paper does not include a systematic meta-analysis or statistical pooling of data, which could provide a more quantitative assessment of ustekinumab’s effectiveness. Although UST has shown a favorable safety profile, data beyond five years are still limited, requiring further long-term observational studies. This review has several limitations that should be acknowledged. The literature search was conducted only in the PubMed database, which may have limited the scope of potentially relevant studies that can be indexed in other scientific databases. Approximately 20% of the initially identified search results were excluded due to a lack of access to full-text articles. This limitation may have led to the omission of relevant data, which could have introduced a bias in the selection of results.

## Figures and Tables

**Table 1:** Short study characteristics of main studies included for qualitative analysis.

Reference	Citation	Year	n	Short Description of the Study	Summary of Results
Ahmed Almradi	[19]	2020	334	Clinical trials of IL-12/IL-23 inhibitors	UST has demonstrated efficacy and safety in treating moderate-to-severe CD. Significant improvements were observed in clinical response, clinical remission, endoscopic, and histologic outcomes, with the 8-week dosing regimen being particularly effective in patients previously treated with biologics.
Laurent Peyrin-Biroulet	[4]	2024	527	Risankizumab versus ustekinumab for moderate-to-severe Crohn’s disease	Risankizumab was non-inferior to ustekinumab in achieving clinical remission at week 24 and superior in endoscopic remission at week 48, with a comparable safety profile between both groups.
Marla Dubinsky	[5]	2023	-	Matching-adjusted indirect comparison between risankizumab and ustekinumab for induction and maintenance treatment	The indirect analysis showed that risankizumab achieved better clinical and endoscopic outcomes than ustekinumab during the induction phase, while CDAI remission in the maintenance phase was comparable.
William J. Sandborn	[20]	2022	-	Five-year efficacy and safety of ustekinumab treatment in Crohn’s disease	UST 90 mg subcutaneously every 12 weeks and every 8 weeks safely maintained clinical response and remission in patients with CD.
Torsten Kucharzik	[31]	2022	77	Early ultrasound response and progressive transmural remission after treatment with ustekinumab	IUS showed that ustekinumab-treated CD patients achieved progressive IUS response (46.3%) and transmural remission (24.1%) through week 48, with a more robust response in the colon and biologic-naive patients.
Sylwio Danese	[32]	2022	498	Assessment that included early endoscopy, regular monitoring of biomarkers and clinical symptoms, and dose intensification	Intensification of ustekinumab treatment based on early endoscopic response, clinical symptoms, and biomarkers did not result in a significant improvement in endoscopic outcomes after 48 weeks compared to standard symptom-based treatment.
Subrata Ghosh	[21]	2024	2575	Safety of ustekinumab in inflammatory bowel disease	The long-term analysis confirms the favorable safety profile of ustekinumab in treating Crohn’s disease with a low incidence of serious adverse events. These findings support the continued use of ustekinumab as a safe therapeutic option for patients with IBD.
Dan Turner	[16]	2024	34	Ustekinumab in pediatric patients-UniStar study long-term extension results	The results confirm the effectiveness and safety of ustekinumab in children with CD, and further studies aim to refine the dosing for children with lower body weight.
Joel R. Rosh	[17]	2021	44	Ustekinumab in pediatric patients with moderately to severely active Crohn’s disease	UST treatment in children improved clinical and endoscopic disease markers as well as inflammatory biomarkers. Pharmacokinetics and safety were consistent with data from adults, and the dosing is appropriate for children weighing ≥40 kg.
Jean-Frédéric Colombel	[23]	2024	915	Evolution of symptoms after ustekinumab induction therapy	Ustekinumab provides rapid symptom relief as early as the first day after infusion, with its efficacy increasing through weeks 16 and 44.
Julian Panes	[33]	2023	440	Ustekinumab improves health-related quality-of-life results up to Week 104	Ustekinumab improved HRQoL, reduced fatigue, and increased patient productivity over 2 years.
Laurent Peyrin-Biroulet	[22]	2024	440	Clinical and endoscopic outcomes with ustekinuma—results from the long-term extension period	The two-year LTE study confirmed the effectiveness of ustekinumab in treating moderate-to-severe Crohn’s disease.
Neeraj Narula	[9]	2022	420	Comparative efficacy and rapidity of action for Infliximab vs. ustekinumab in biologic naïve Crohn’s disease	Infliximab and ustekinumab show comparable efficacy and rate of action
Emily C.L. Wong	[34]	2023	220	Comparative efficacy of Infliximab vs. ustekinumab for maintenance of clinical response	Infliximab may give better results in terms of endoscopic outcomes.
Neeraj Narula	[6]	2021	941	The effect of ustekinumab on extraintestinal symptoms of Crohn’s disease	UST does not reduce the risk of new EIMs in the short term but improves their course in the long term, especially in arthritis and erythema nodosum.
Emily C.L. Wong	[26]	2021	254	Randomized controlled trial about BMI and clinical efficacy of UST	After 308 days of treatment, UST levels were significantly lower in obese individuals, but no correlation was found between BMI and treatment effectiveness.
Daniel C. Baumgart	[7]	2024	52	Clinical trial about UST and mucosal healing	Among the patients, 52% achieved a combined clinical and endoscopic response, and 15 patients achieved an endoscopic response. Adverse events occurred in 69.2% of patients, among which 15.4% were classified as severe, but no serious events or deaths were observed.
Neeraj Narula	[36]	2024	373	Ustekinumab and adalimumab	The clinical response after one year of treatment with both drugs was similar in the group of patients who responded early and those with a delayed response.
Bruce E. Sands	[8]	2022	386	Ustekinumab versus adalimumab	In this group of patients who had not been previously treated with biologics, both ustekinumab and adalimumab in monotherapy demonstrated high efficacy, with no differences observed in the primary treatment outcome between these drugs.
Jia-yin Yao	[18]	2021	20	Ustekinumab trough concentration affects treatment	The optimal minimum concentration of UST was determined to be 1.12 μg/mL. Patients with UST levels exceeding 1.12 μg/mL demonstrated better clinical response outcomes.
Vincent Chow	[30]	2023	238	Randomized controlled trial about ABP 654, an ustekinumab biosimilar candidate	Study may contribute to the globalization of CD therapy with ABP 65.
Sigrid Balser	[35]	2024	491	FYB202, an ustekinumab biosimilar candidate	FYB202 has pharmacokinetic parameters comparable to EU/US USTs.
Emily C.L. Wong	[24]	2023	683	Predictors of CD clinical remission	Inclusion of people with shorter disease duration may increase the effectiveness of therapy and improve interpretation of results.
Emily C.L. Wong	[25]	2025	683	The role of eosinophils as a potential biomarker of response to ustekinumab	Those responding positively to therapy had significantly higher initial eosinophil counts.
Tursi	[28]	2021	194	Ustekinumab as second- or third-line therapy in Crohn’s disease	UST is regarded as an effective and safe second-line therapy for CD patients refractory to other biologic treatments.
Mark T. Osterman	[27]	2021	106	Epithelial cell biomarkers	MVL may be an important biomarker for predicting the response to UST treatment.
William J. Sandborn	[29]	2021	2574	Safety of ustekinumab	UST has a favorable safety profile in the treatment of inflammatory bowel disease.
Matthieu Allez	[37]	2023	243	Safety of ustekinumab	UST was associated with a safe action profile compared to Tesnatilimab.
Vince B. C. Biemans	[38]	2020	221	Observational study about UST and CD	Ustekinumab is a safe and effective treatment option for CD patients who did not respond to prior TNF and integrin inhibitor therapies.
Anders Forss	[39]	2023	114	UST long-term effectiveness and improved health-related quality of life	The study confirms the long-term effectiveness and safety of ustekinumab in treating CD.
Tessa Straatmijer	[40]	2021	252	Safety of ustekinumab	Approximately one in three patients with CD achieved clinical remission.
Maria Chaparro	[41]	2022	463	Long-term real-world effectiveness and safety	UST safe as short- and long-term treatment.
Marisa Iborra	[42]	2020	407	Long-term safety of UST	UST proved effective in achieving both clinical and endoscopic remission.

n—Number of participants in the study, CDAI—The Crohn’s Disease Activity Index, IUS—Non-invasive imaging methods—intestinal ultrasonography, BMI—body mass index, UST—Ustekinumab, MVL—Microvesicle lengths of the ileum, HRQoL—Health-related quality of life, EIM—Ex-Extraintestinal manifestations.

**Table 2:** Key study characteristics and findings of UST in CD.

Subsection	Main Findings and Conclusions with Citations
3.1 Efficacy of ustekinumab in the treatment of CD	Clinical/endoscopic response was achieved in 2%; Significant predictors: age. Fistula healing, intestinal manifestations dropped 20.2% at 52 weeks [7]. UST induces endoscopic remission, especially after multiple biologics [7]. UST levels ≥ 1.12 μg/mL linked to better outcomes [18]. EIMs reduced 35% (week 6) and 72% (week 52) [6]. Higher remission rates in UST groups vs. placebo (UNITI trials) [19]. Long-term remission with UST (IM-UNITI study): clinical remission 28.7% (q12w), 34.4% (q8w) after 5 years [20]. STARDUST: 50.2% clinical remission, 39.3% endoscopic response at 104 weeks [22]. Colombel JF: Higher remission in early-phase UST therapy [23].
3.2 Predictive factors of response to therapy	Shorter disease duration (<5 years for remission, <1 year for clinical response) and higher eosinophil counts predict positive response [24]. UST levels ≥ 1.12 μg/mL improve endoscopic remission [18]. BMI impacts UST drug levels, but not treatment success [26]. Ileal microvessel length > 1.7 µm correlates with higher efficacy [27].
3.3 Clinical implications of UST therapy	Symptoms improved in 75% at 4 months and 70% at 6 months. Endoscopic symptom improvement in 50%. CRP and fecal calprotectin decreased, FC > 200 µg/g correlated with lack of remission [28]. Safety profile: TEAEs in 69.2%, 15.4% severe but no deaths [7]. Common TEAEs: infections, headache, fatigue, nasopharyngitis [29]. UST response and trans-wall remission observed (STARDUST) [31]. HRQoL and productivity improved with UST, no significant difference between T2T and SoC [33]. At week 104, 50.2% clinical remission, 39.3% endoscopic response. Flexible dosing maintained efficacy [22].
3.4 Quality of life in patients undergoing UST therapy	Ustekinumab significantly improves quality of life in Crohn’s disease patients, as shown in adult (STARDUST) and pediatric (UniStar) studies, through symptom reduction, decreased fatigue, and better daily and social functioning.
3.5.1 UST and adalimumab	Clinical remission was 4% greater in UST vs. adalimumab. Endoscopic lesion improvement similar in both groups [8]. Early and late responders had comparable outcomes [25].
3.5.2 UST and risankizumab	UST no worse than RZB at 24 weeks; RZB superior at 48 weeks for endoscopic remission [4]. RZB superior in inducing endoscopic remission after prior biologic failure [5].
3.5.3 UST and infliximab	No significant differences in clinical remission between UST and infliximab [9]. Infliximab better for endoscopic response and remission [34].
3.5.4 UST and biosimilars substitutes	ABP 65 shows fewer ADA antibodies compared to UST, with a similar safety profile [30]. FYB202 had comparable safety and pharmacokinetics to UST [30,35].
3.6 Ustekinumab in pediatric patients	UniStar study: UST improved efficacy and serum levels in children [16,17]. Long-term UST treatment improved clinical remission, quality of life, and biomarkers [16].

CD—Crohn’s disease, CRP—C-reactive protein, FC—Fecal calprotectin, TEAEs—Treatment-emergent adverse events, UST—Ustekinumab, ADA—Anti-drug antibodies, HRQoL—Health-related quality of life, T2T—Treat-to-target, SoC—Standard of care, RZB—Risankizumab.

**Table 3:** Summary of the adverse events in studies included in the narrative review.

Adverse Event	Study	n	n%
Discontinuation of treatment in the study due to adverse events	[4] Laurent Peyrin-Biroulet [16] Dan Turner [17] Joel R. Rosh [22] Laurent Peyrin-Biroulet [8] Bruce E. Sands [28] Tursi	13 5 2 13 5 4	4.9 14.7 5 4 6 2
Serious adverse events	[4] Laurent Peyrin-Biroulet [16] Dan Turner [8] Bruce E. Sands [30] Vincent Chow [29] William J. Sandborn	46 11 25 1 70	17.4 32.4 13 1.3 4.4
Infections	[8] Bruce E. Sands [16] Dan Turner [17] Joel R. Rosh [22] Laurent Peyrin-Biroulet [29] William J. Sandborn [41] Maria Chaparro	65 25 17 45 305 5	34 73.5 39 13.9 19.3 1.1
Serious infections	[4] Laurent Peyrin-Biroulet [16] Dan Turner [17] Joel R. Rosh [8] Bruce E. Sands [30] Vincent Chow [29] William J. Sandborn	11 0 0 4 2 18	4.2 0 0 2 2.5 1.1
Nasopharyngitis	[32] Sylwio Danese [22] Laurent Peyrin-Biroulet [8] Bruce E. Sands [30] Vincent Chow [35] Sigrid Balser [29] William J. Sandborn	29 28 14 2 101 78	13 8.7 7 2.5 20.6 4.9
Abdominal pain	[32] Sylwio Danese [22] Laurent Peyrin-Biroulet [8] Bruce E. Sands [30] Vincent Chow	23 22 24 1	11 6.8 13 1.3
Joint pain	[22] Laurent Peyrin-Biroulet [8] Bruce E. Sands [29] William J. Sandborn	20 12 69	6.2 6 4.4
Upper respiratory tract infection	[8] Bruce E. Sands [29] William J. Sandborn	12 44	6 2.8
Rash at the injection site	[8] Bruce E. Sands	3	2
Vomiting	[8] Bruce E. Sands [30] Vincent Chow [29] William J. Sandborn	10 0 41	5 0 2.6
Diarrhea	[8] Bruce E. Sands [35] Sigrid Balser	11 17	6 3.5
Hypersensitivity	[4] Laurent Peyrin-Biroulet [41] Maria Chaparro	24 9	9.1 1.9
Liver events	[4] Laurent Peyrin-Biroulet	14	5.3
Headache	[32] Sylwio Danese [17] Joel R. Rosh [22] Laurent Peyrin-Biroulet [35] Sigrid Balser [29] William J. Sandborn [41] Maria Chaparro	24 8 24 142 106 7	11 18 7.4 28.9 6.7 1.5
Anemia	[17] Joel R. Rosh	7	16
COVID-19	[35] Sigrid Balser	36	7.3
Fever	[29] William J. Sandborn [41] Maria Chaparro	58 1	3.7 0.2

n—number of subjects in the study taking UST, n%—% of subjects taking the drug.

**Table 4:** Comparison of UST treatment in adults vs. pediatric patients with CD.

Category	Adults	Pediatric Patients
Induction regimen	Intravenous: standard dose of 6 mg/kg body weight.	Intravenous: 130 mg or 390 mg for ≥40 kg; 3 mg/kg or 9 mg/kg for <40 kg (UniStar study).
Maintenance regimen	Subcutaneous: 90 mg every 8 or 12 weeks, depending on clinical response.	Subcutaneous: 90 mg (≥40 kg) or 2 mg/kg (<40 kg), starting from week 8.
Efficacy	Confirmed in several studies (e.g., UNITI-1, UNITI-2, IM-UNITI); clinical remission and biomarker improvement achieved.	In the UniStar study: clinical improvement and reduction in inflammatory biomarkers by week 8; remission achieved in 41.2% of patients at week 48.
Long-term efficacy	Maintained efficacy over years with regular treatment, supported by observational data.	Long-term treatment (up to 240 weeks) resulted in sustained remission and improved quality of life (Turner D. et al.).
Drug levels	Stable, predictable pharmacokinetics.	Children < 40 kg had lower serum drug levels—suggesting the need for dose adjustment.
Safety	Favorable safety profile; rare serious adverse events; low immunogenicity.	Good safety profile. Most common serious adverse event: CD exacerbation (n = 6); only one case of anti-UST antibodies.
Immunogenicity (antibodies)	Low incidence of anti-UST antibodies in adult population.	No significant anti-UST antibodies detected, except in one case.

UST—Ustekinumab, CD—Crohn’s disease, mg—milligram, kg—kilogram, UniStar—Name of the clinical trial in the pediatric population, UNITI-1, UNITI-2, IM-UNITI—Names of clinical trials conducted in adults with CD.

## Data Availability

No new data were created or analyzed in this study.

## Abbreviations

The following abbreviations are used in this manuscript:

UST	Ustekinumab
EN	Erythema nodosum
BMI	Body mass index
EIM	Extraintestinal manifestations
CD	Crohn’s disease
IBDs	Inflammatory bowel diseases
CR	Clinical remission
CRP	C-reactive protein
CDAIs	The Crohn’s Disease Activity Index
(STRIDE) II	Selecting Therapeutic Targets in Inflammatory Bowel Disease
RZB	Risankizumab
WPAI	Activity impairment
HRQoL	Health-related quality of life
IUS	non-invasive imaging methods—intestinal ultrasonography
SES-CD	Simple endoscopic score for Crohn’s disease
NGNA	N-glycolylneuraminic acid
ADA	Antidrug antibody
SoC	Standard of care
T2T	Treat-to-target
LTE	Long-term extension
MVL	Intestinal microvilli length
q12w	Every 12 weeks
q8w	Every 8 weeks
TNFα	Tumor necrosis factor alpha
GUS	Guselkumab
TIL	Tildrakizumab
AE	Adverse event
